# Supplementary material for: Maintenance of neuronal TDP-43 expression requires axonal lysosome transport
Source: bioRxiv. 2024 Oct 1:2024.09.30.615241. Preprint. [Version 1] doi: 10.1101/2024.09.30.615241 (PMC11722429; doi:10.1101/2024.09.30.615241)
Supplement: Supplementary file 1 [file media-1.pdf]

Supplemental figures and legends

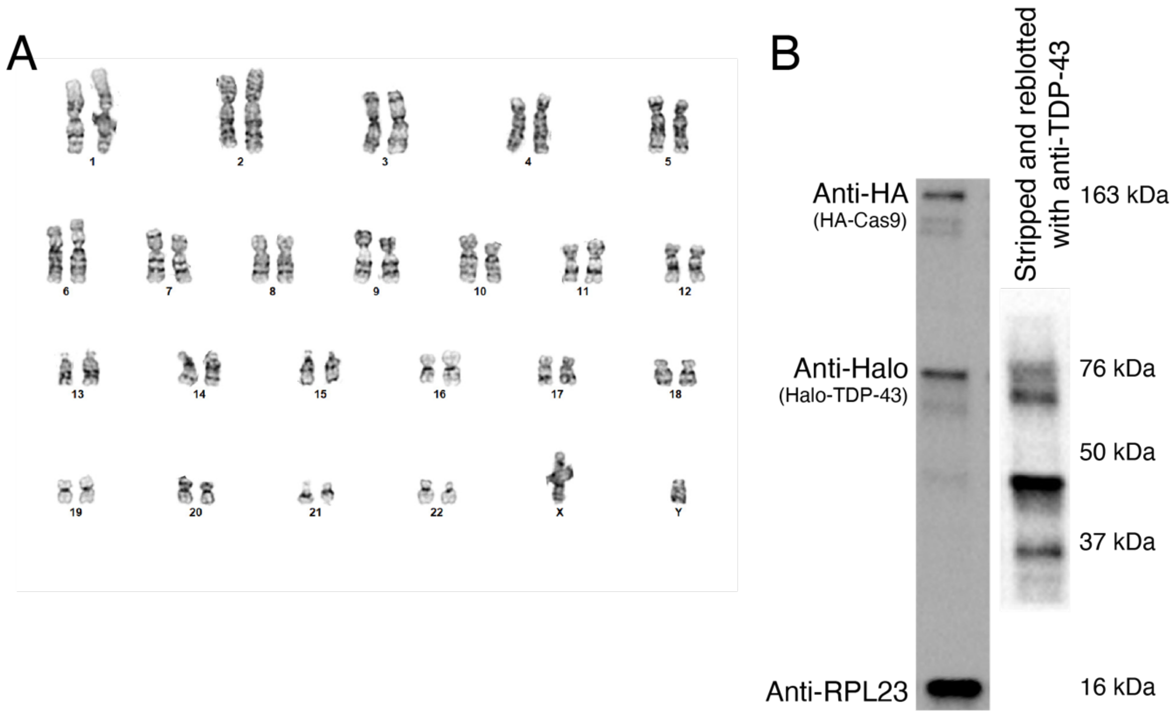

Figure S1 (related to Figure 1): i11w-hT iPSCs have a normal karyotype.

A) Left blot shows Cas9, Halo-TDP-43, and RPL23 staining (Anti-HA, Anti-Halo, and Anti-RPL23, respectively), demonstrating TDP-43 is HaloTagged. On right, blot was stripped and re-probed with anti-TDP-43, showing both HaloTagged TDP-43 and untagged TDP-43.

B) Karyotype of Halo-TDP-43 iPSCs shows a normal karyotype (44 chromosomes + XY), demonstrating that CRISPR editing did not cause any chromosomal abnormalities.

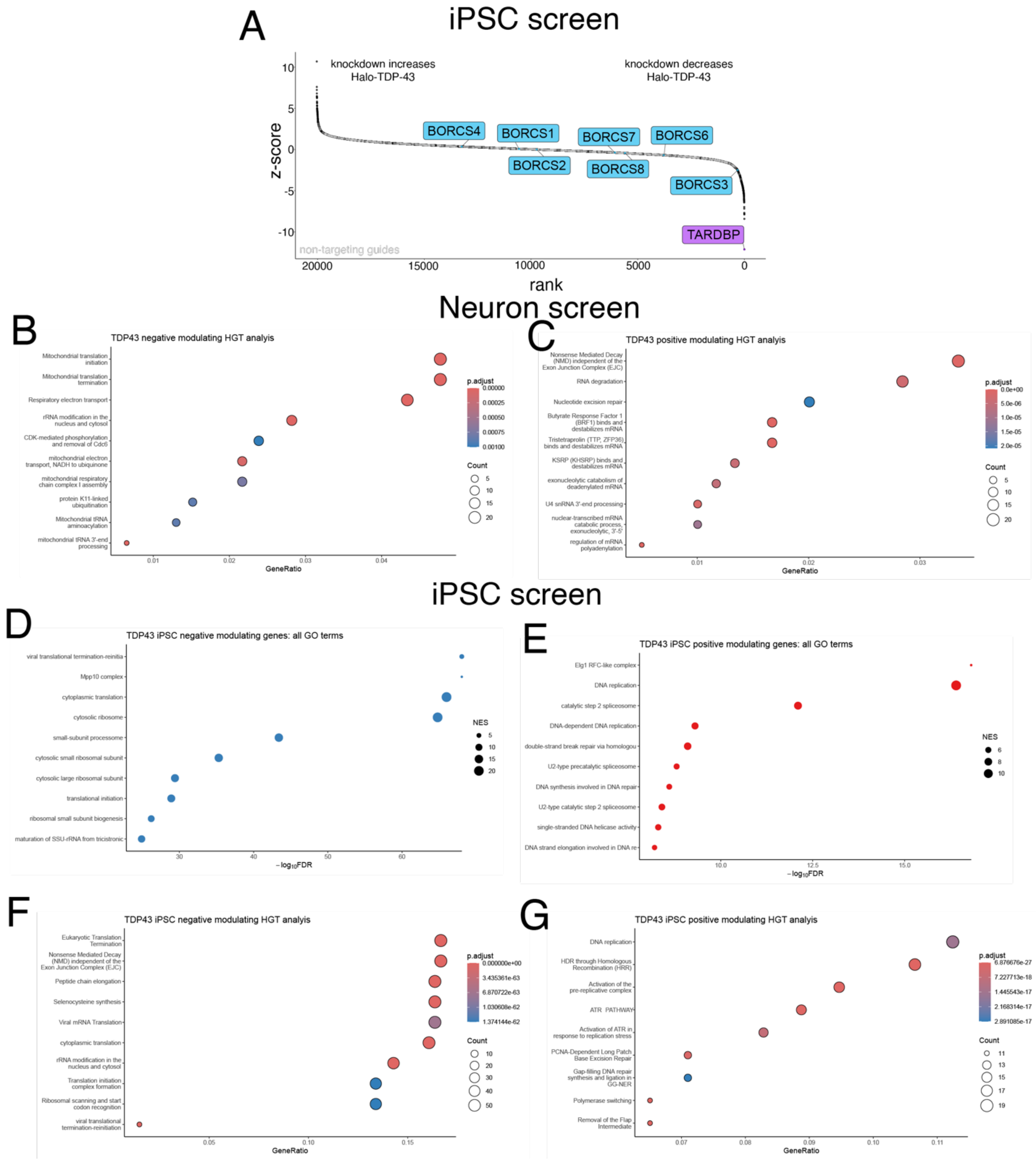

Figure S2 (related to Figure 2): iPSC screen shows different hits than neuron screen.

A) Rank plot of iPSC screen results showing genes whose KD increases Halo-TDP-43 levels (left) and genes whose KD decreases Halo-TDP-43 levels, including TDP-43 itself (right). BORC genes are indicated in blue.
B) HGT analysis of screen hits that decrease Halo-TDP-43 levels in neurons also shows many mitochondrial terms, as was seen in the GO analysis.
C) HGT analysis of screen hits that increase Halo-TDP-43 levels in neurons shows many RNA metabolism hits.

D) GO analysis of hits that decrease Halo-TDP-43 levels in iPSCs shows many ribosome and translation-related terms, a striking difference from the hits found in neurons. The FDR is from the calculated permutation p value of 1000 iterations.
E) GO analysis of hits that increase Halo-TDP-43 levels in iPSCs shows DNA synthesis, spliceosome, and DNA repair terms. The FDR is from the calculated permutation p value of 1000 iterations.
F) HGT analysis of hits that decrease Halo-TDP-43 levels in iPSCs shows translation, ribosome, and NMD-related terms. The FDR is from the calculated permutation p value of 1000 iterations.
G) HGT analysis of hits that increase Halo-TDP-43 levels in iPSCs shows DNA related terms. The FDR is from the calculated permutation p value of 1000 iterations.

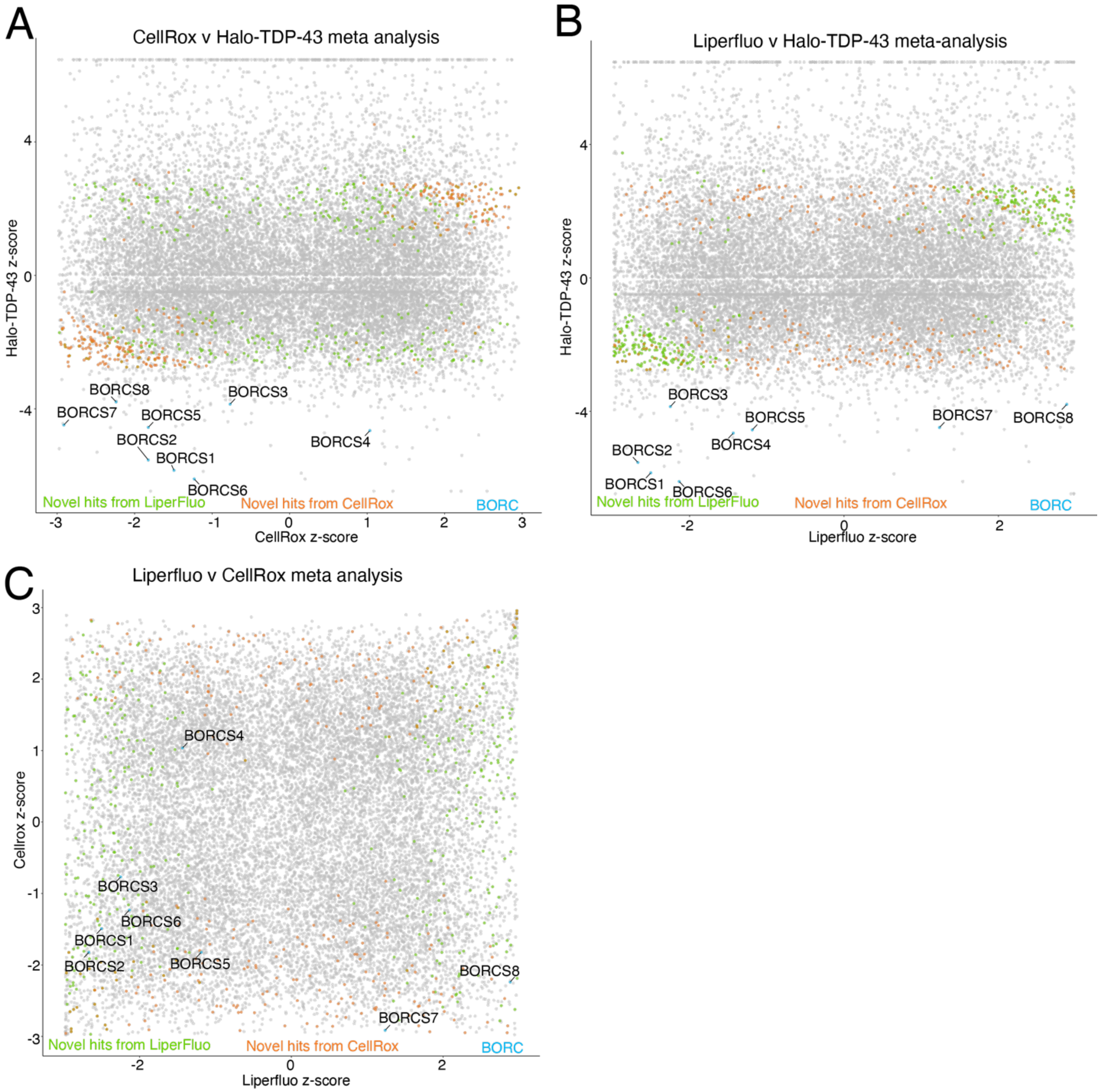

Figure S3 (related to Figure 3): Meta-analyses of Halo-TDP-43 and published CRISPRi FACS screens.

A) Comparison between CellRox and Halo-TDP-43 meta-analysis z-scores with novel hits highlighted. BORC genes are highlighted in blue.
B) Comparison between Liperfluo and Halo-TDP-43 meta-analysis z-scores with novel hits highlighted. BORC genes are highlighted in blue.
C) Comparison between CellRox and Liperfluo meta-analysis z-scores with novel hits highlighted. BORC genes are highlighted in blue.

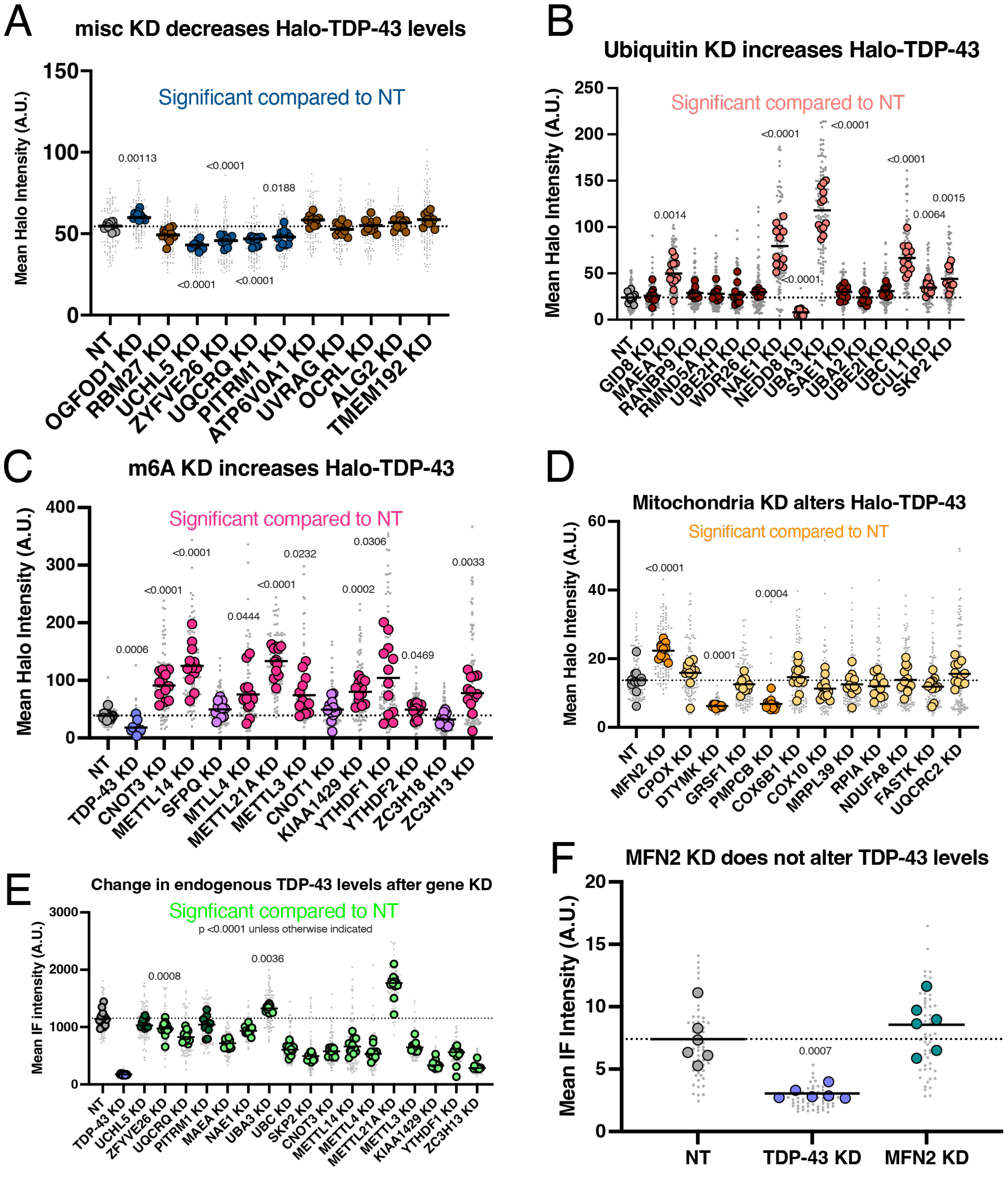

Figure S4 (related to Figure 4): Validation of neuron screen results show many genes alter TDP-43 levels.

A) Quantification of miscellaneous KD Halo-TDP-43 microscopy. OGFOD1 KD increases Halo-TDP-43 levels while UCHL5 KD, ZYFVE26 KD, UQCRQ KD, and PITRM1 KD decrease Halo-TDP-43 levels compared to a non-targeting (NT) guide, indicated by dark blue dots. N=12 wells per genotype, 9 images per well (small grey dots). Significant p-values indicated on graph.
B) Quantification of ubiquitin-associated gene KD Halo-TDP-43 microscopy. MAEA, NAE1, UBA3, UBC, CUL1, and SKP2 KD all increase Halo-TDP-43 levels compared to a non-targeting (NT) guide, indicated by orange dots. NEDD8 KD decreases Halo-TDP-43 levels due to a strong survival phenotype. N=12 wells per genotype, 9 images per well (small grey dots). Significant p-values indicated on graph.
C) Quantification of m6A-associated gene KD Halo-TDP-43 microscopy. CNOT3, METTL14, MTLL4, METTL21A, METTL3, KIAA1429, YTHDF2, and ZC3H13 KD all increase Halo-TDP-43 levels compared to a non-targeting (NT) guide, indicated by magenta dots. N=12 wells per genotype, 9 images per well (small grey dots). Significant p-values indicated on graph.
D) Quantification of mitochondria-associated gene KD Halo-TDP-43 microscopy. MFN2 KD increases Halo-TDP-43 levels while DTYMK and PMPCB KD decrease Halo-TDP-43 levels compared to a non-targeting (NT) guide, indicated by dark blue dots. N=12 wells per genotype, 9 images per well (small grey dots). Significant p-values indicated on graph.
E) Quantification of TDP-43 immunofluorescence (IF) microscopy. TDP-43 KD significantly decreases TDP-43 levels by IF. ZFYVE26, UQCRQ, , MAEA, NAE1, UBC, SKP2, CNOT3, METTL14, METTL4, METTL3, KIAA1429, YTHDF1, and ZC3H13 KD all decrease TDP-43 IF levels (although not all decrease Halo-TDP-43 levels), while UBA3 and METTL21A KD increase TDP-43 IF levels, indicated by lime dots. N=12 wells per genotype, 9 images per well (small grey dots). Significant p-values indicated on graph.
F) Quantification of MFN2 KD TDP-43 immunofluorescence microscopy. MFN2 KD does not significantly change untagged TDP-43 levels compared to a NT guide, indicated by teal dots, while TDP-43 KD does significantly decrease TDP-43 IF levels. N=6 wells per genotype, 9 images per well (small grey dots). Significant p-values indicated on graph.

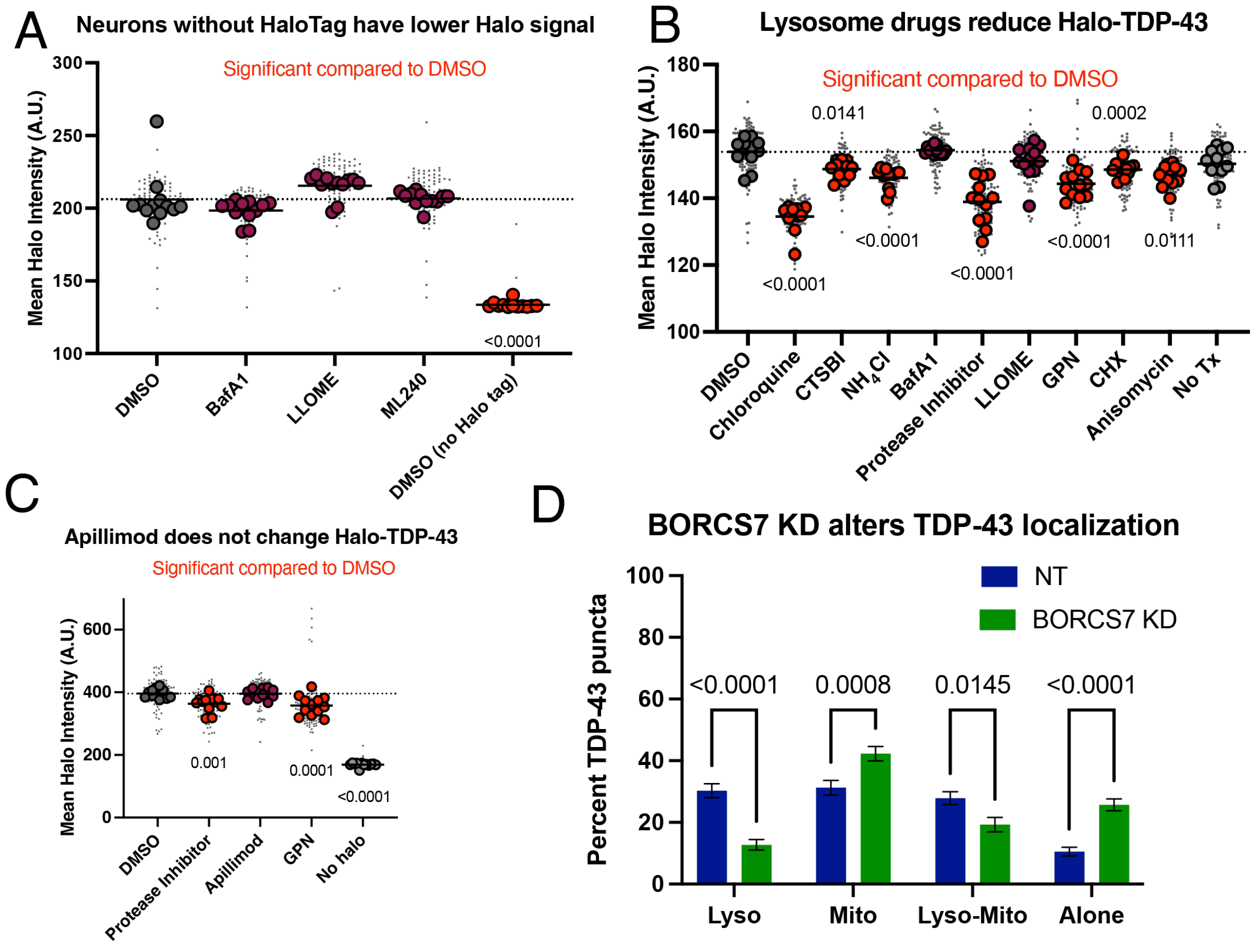

Figure S5 (related to Figure 6): Lysosome inhibitors alter Halo-TDP-43 levels.

A) Additional drug treatments from experiment shown in Figure 5B that do not change Halo-TDP-43 levels. An untagged control line (“no Halo tag”) shows significantly less Halo signal than the DMSO control (repeated from Figure 5B).
B) Replication of drug treatment experiment shows the same trend as previous experiment.
C) Apillimod does not alter levels of Halo-TDP-43.
D) Quantification of Halo-TDP-43-organelle association after BORCS7 KD. Halo-TDP-43 is significantly reduced on lysosomes, with a small increase of Halo-TDP-43 on mitochondria. More Halo-TDP-43 is transported not on either lysosomes or mitochondria in BORCS7 KD neurons.

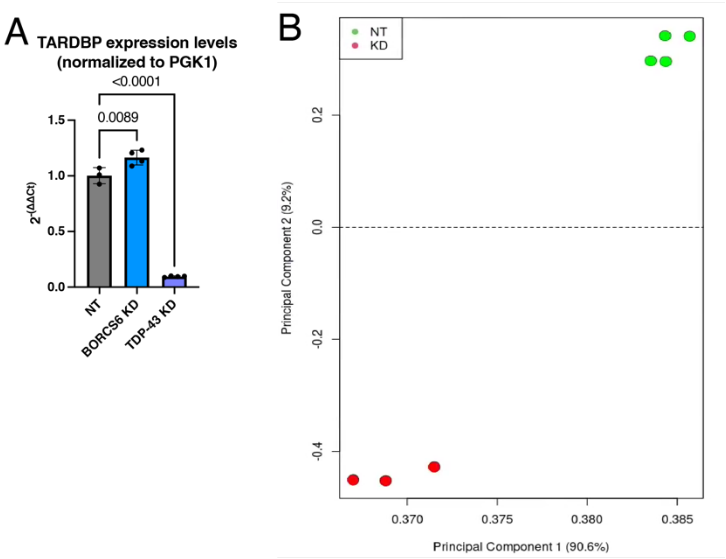

Figure S6 (related to Figure 7): TDP-43 mRNA levels are unchanged after BORCS6 KD.

A) qPCR of NT, BORCS6 KD, and TDP-43 KD neurons showing decreased TDP-43 mRNA levels in TDP-43 KD, and slight, but significant increase in TDP-43 mRNA in BORCS6 KD. Normalized to PGK1. NT n=3, BORCS6 KD n=4, and TDP-43 KD n=4.

B) Principal component analysis plot of RNA sequencing data shows most of the variance between the samples (90%) can be explained by a single principal component and that the samples separate based on knockdown status.

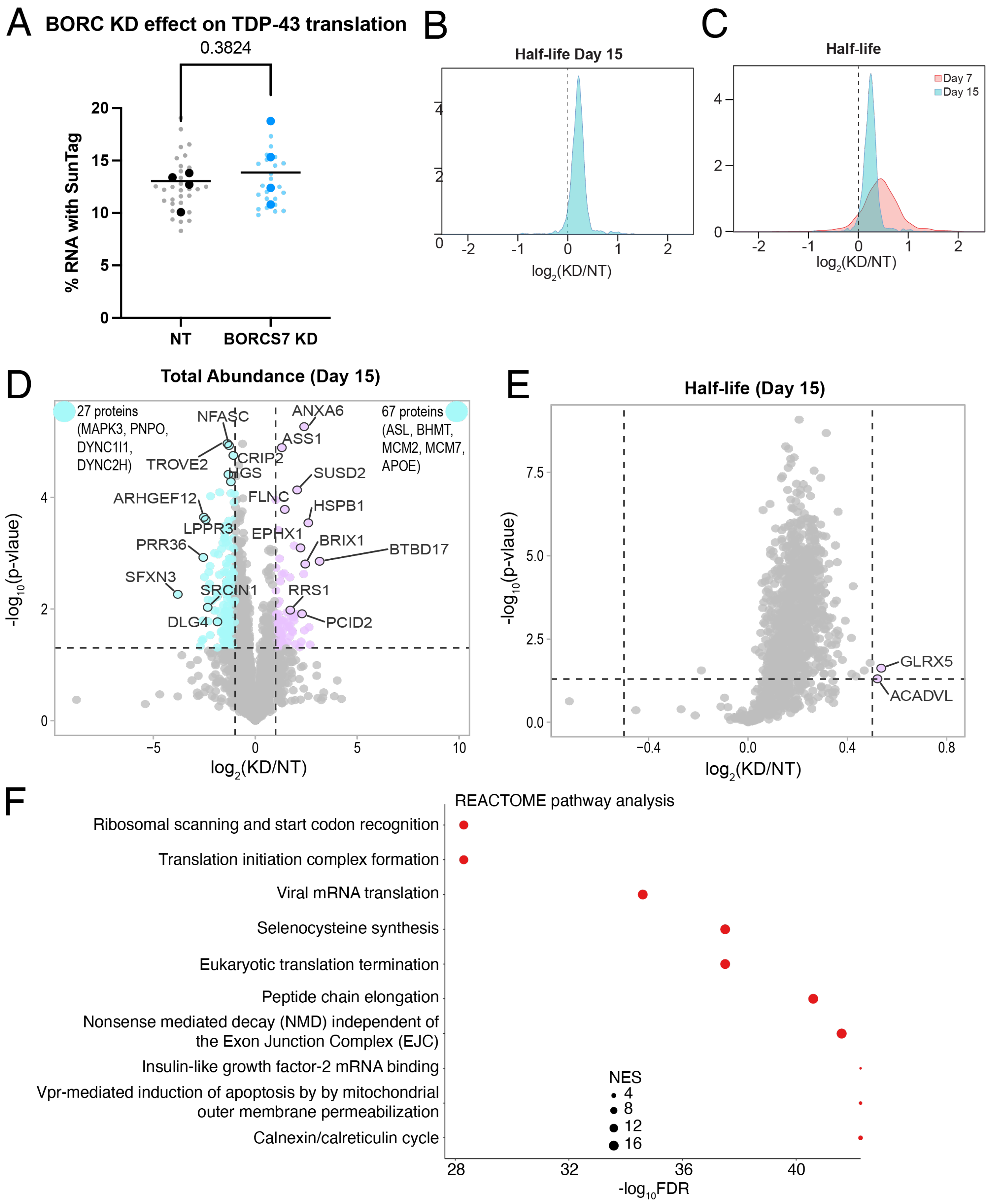

Figure S7 (Related to figure 8): Protein turnover is increased at d15 in BORC KD neurons.

A) TDP-43 translation is not altered upon BORCS7 KD, as measured by fraction of RNA puncta with SunTag puncta by microscopy. N=4 wells, 8 images quantified per well.

B) Density plot of total protein half-life shows longer protein half-lives in BORCS6 KD neurons compared to NT neurons at d15.

C) Overlay of protein half-life density plots at d7 (red) and d15 (teal) show both days have longer protein half-lives in BORCS6 KD neurons.
D) Volcano plot of total protein abundance comparing BORCS6 KD to a non-targeting guide at d15. Some proteins (purple) have increased abundance, while a larger number (teal) have decreased abundance. Non-significant or low log_2(fold change) proteins are indicated in grey. Vertical lines at log_2(fold change) of 1 and -1, horizontal line at p-value = 0.05.
E) Volcano plot of total protein half-life at d15 show many proteins have longer half-lives (log_2(fold change) > 0) in BORCS6 KD neurons as compared to neurons expressing a non-targeting guide. Non-significant or low log_2(fold change) protein turnovers are indicated in grey. Horizontal line at p=0.05, vertical lines at log_2(fold change) of 0.5 or -0.5.
F) Gene ontology enrichment analysis using proteins with longer half-lives in BORCS6 KD neurons shows REACTOME pathways related to ribosomes, translation, and mRNA binding.

Supplementary table legends
Table S1: Individual guide sequences cloned for screen validation.

Table S1: Individual guide sequences cloned for screen validation.

Gene	guide sequence	Ward lab #	selection	color
BLOC1S1_1	GGGGACAGCATGGTCACGTC	SG1021	puro	BFP; cytosolic
BLOC1S1_2	GGAAGCTGGAACGCTCACCT	SG1022	puro	BFP; cytosolic
BLOC1S2_1	GAACAGCGCGGGGTCCGCTA	SG1023	puro	BFP; cytosolic
BLOC1S2_2	GAGCGCGGGGTCCGCTATGG	SG1024	puro	BFP; cytosolic
SNAPIN_1	GGTCCCTGCCCCCGATACAG	SG1025	puro	BFP; cytosolic
SNAPIN_2	GGGTTCCGCCGCTGTATCGG	SG1026	puro	BFP; cytosolic
LOH12CR1_1	GCTCCTTCCGGGACGCAGAT	SG1027	puro	BFP; cytosolic
LOH12CR1_2	GGGCCGGGCGGTCACCGCTC	SG1028	puro	BFP; cytosolic
C17orf59_1	GGGGCGGCCCGGGCCCGAGA	SG1029	puro	BFP; cytosolic
C17orf59_2	GGACTCCATACTGCAGGTGG	SG1030	puro	BFP; cytosolic
KXD1_1	GctctcaaaatgttgattCC	SG1031	puro	BFP; cytosolic
KXD1_2	GGCCAGACTAGCAGTAATCA	SG1032	puro	BFP; cytosolic
MEF2BNB_1	GGGGCGTCTGGAACTTAAAG	SG1033	puro	BFP; cytosolic
MEF2BNB_2	GGGCTCCTCCATAGCGACCG	SG1034	puro	BFP; cytosolic
C10orf32_1	GAAAGGCTCCCGGAGCTCCG	SG1035	puro	BFP; cytosolic
C10orf32_2	GCGGTTCGGTCAGTCCGTGA	SG1036	puro	BFP; cytosolic
OGFOD1	GTCAAGACGCTACCTTCCTG	SG1037	puro	BFP; cytosolic
RBM27	GGCTGTGAAGGGAACGTGAG	SG1038	puro	BFP; cytosolic
UCHL5	GGACGTGTGGTTGACGAGCT	SG1039	puro	BFP; cytosolic
ZFYVE26	GGACGAATCTGTGTCATAAG	SG1040	puro	BFP; cytosolic
UQCRQ	GACCGGATCGGTGACTGTGG	SG1041	puro	BFP; cytosolic
PITRM1	GGGCGGGTGAGACGCTGCGA	SG1042	puro	BFP; cytosolic
ATP6V0A1	GGCCAAACCCGCACCCTCCG	SG1043	puro	BFP; cytosolic
UVRAG	GGCTCTTCCTTAGCcagcgg	SG1044	puro	BFP; cytosolic
OCRL	GCGAGGCGCCGCTCTCTCTT	SG1045	puro	BFP; cytosolic
ALG2	GGGTGGAGGAACAGCACCGA	SG1046	puro	BFP; cytosolic
TMEM192	GCGTGAGTGCACGGAGGCGG	SG1047	puro	BFP; cytosolic
GID8	GGGGGCGCCCGAGTAGAAgg	SG1059	puro	BFP; cytosolic
MAEA	GAAGATGGCGGTGCAGGAGT	SG1060	puro	BFP; cytosolic
RANBP9	GGAAACAGCCGCGAGAGCAG	SG1061	puro	BFP; cytosolic
RMND5A	GCTGCAGGATCTCGTGCTTG	SG1062	puro	BFP; cytosolic
UBE2H	GGGCAACACCCCCGCGGTCC	SG1063	puro	BFP; cytosolic
WDR26	GCCTCGGCGGCGGAGGGAGT	SG1064	puro	BFP; cytosolic
NAE1	GTGGTGGCGGCGCTCCGCAG	SG1065	puro	BFP; cytosolic
NEDD8	GCAGCGGCGACCGGAGCGGT	SG1066	puro	BFP; cytosolic
UBA3	GCGAGGAGCCGTAAGTACCG	SG1067	puro	BFP; cytosolic
PIAS1	GGCGGACAGTGCGGAACTAA	SG1068	puro	BFP; cytosolic
SAE1	GCGGTTGGCTTGAGCGGGAC	SG1069	puro	BFP; cytosolic
UBA2	GGGACAACCACGAGCCGCGG	SG1070	puro	BFP; cytosolic
UBE2I	GAGGGAAGTCCCGAGACAAA	SG1071	puro	BFP; cytosolic
PSMB3	GGCCCCGGTGAGGACCAAGA	SG1072	puro	BFP; cytosolic
UBC	GTATAAGGACGCGCCGGGTG	SG1073	puro	BFP; cytosolic
CUL1	GGGTTGCGTGGTGCAGGCGG	SG1074	puro	BFP; cytosolic
SKP2	GCTTGCGCGCGCAGTGGGGA	SG1076	puro	BFP; cytosolic

CNOT3	GGTCGCGATACGCTCCTCAG	SG1084	puro	BFP; cytosolic
METTL14	GGCGCCGGAAGTCTCTACTG	SG1085	puro	BFP; cytosolic
SFPQ	GAGACGCTCAGGAAACGTGG	SG1086	puro	BFP; cytosolic
METTL4	GAGGGCGAGTTGAGCGTTCA	SG1087	puro	BFP; cytosolic
METTL21A	GCCTCCCGGGCCGTAAGTAC	SG1088	puro	BFP; cytosolic
METTL3	GTGGGGCCGAGGCTCAACCG	SG1089	puro	BFP; cytosolic
CNOT1	GCTCCGGGAAACGCTTCCAG	SG1090	puro	BFP; cytosolic
KIAA1429	GACGCGATGCTCACACAGCT	SG1091	puro	BFP; cytosolic
YTHDF1	GTGACAGGCCCGGCCTCTAG	SG1092	puro	BFP; cytosolic
YTHDF2	GGCAGAGCGCCGAACAGCGG	SG1093	puro	BFP; cytosolic
ZC3H18	GGCTCCAGGCTCGACACGGG	SG1094	puro	BFP; cytosolic
ZC3H13	GTAGCCAAGAGCCGGTTCCC	SG1095	puro	BFP; cytosolic
MFN2	GCGCGAGGCCGGGAAGGTGA	SG1136	Puro	BFP; cytosolic
CPOX	GTCGCCGGCTCAATACTCCG	SG1137	Puro	BFP; cytosolic
DTYMK	GCGCGGGGCTCTCATAGTGC	SG1138	Puro	BFP; cytosolic
GRSF1	GTGCCGGCCATGGACTCCGG	SG1139	Puro	BFP; cytosolic
PMPCB	GCGCCACAGATCCGAACAGT	SG1140	Puro	BFP; cytosolic
COX6B1	GTACCGGCGAGATCTCTACC	SG1141	Puro	BFP; cytosolic
COX10	GCAGCGTCCCGTGAGGAGAG	SG1142	Puro	BFP; cytosolic
MRPL39	GACGGTTCTCACCGCTGCTA	SG1143	Puro	BFP; cytosolic
RPIA	GGTGCACAGTCTGGGACCCG	SG1144	Puro	BFP; cytosolic
NDUFA8	GGGCTGAGGGCTTCTCGTCG	SG1145	Puro	BFP; cytosolic
FASTK	GCCCCCCGCGGCCTCCTCAT	SG1146	Puro	BFP; cytosolic
UQCRC2	GCCCGCAGGGTTCAGCTGAA	SG1147	Puro	BFP; cytosolic
Nontargeting_control_1872	GTCCACCCTTATCTAGGCTA	SG100	Puro	BFP; cytosolic
TDP-43	GGGAAGTCAGCCGTGAGACC	SG200	Puro	BFP; cytosolic
C10orf32_1_p-lentiCRISPR	CGCGATTACGTCAGTACCAC	SG1187	Puro	BFP; cytosolic
C10orf32_2_p-lentiCRISPR	GATTACGTCAGTACCACAGG	SG1188	Puro	BFP; cytosolic
non-targeting_p-lentiCRIPSR	AGAGTTACGTCGCTTCGATC	SG1190	Puro	BFP; cytosolic
